# Through the Looking Glass: A Scoping Review of Cinema and Video Therapy

**DOI:** 10.3389/fpsyg.2021.732246

**Published:** 2022-01-11

**Authors:** Elena Sacilotto, Gerardo Salvato, Federica Villa, Fulvia Salvi, Gabriella Bottini

**Affiliations:** ^1^Department of Brain and Behavioral Sciences, University of Pavia, Pavia, Italy; ^2^Cognitive Neuropsychology Centre, ASST “Grande Ospedale Metropolitano” Niguarda, Milan, Italy; ^3^NeuroMi, Milan Center for Neuroscience, Milan, Italy; ^4^Department of Human Studies, University of Pavia, Pavia, Italy; ^5^Medicinema Italia Onlus, Milan, Italy

**Keywords:** cinematherapy, video modeling, video peer-modeling, video therapy, art therapy

## Abstract

**Background:** Cinematherapy and video treatments are artistic therapeutic techniques by which the individuals are exposed to their psycho-physical difficulties through the stories of the characters on the screen who are coping with the same issues that the patients are. Although these techniques are increasingly common within modern art therapies, there are neither comprehensive classifications of the different approaches nor agreement on their effectiveness. We performed a scoping review, describing different methodological approaches and outcome measures in cinematherapy and video treatments.

**Methodology:** We searched articles in PubMed, PsycINFO and Google Scholar. We included: (i) articles in which subjects were treated for their difficulties with videos or films, (ii) articles written in English. Review articles and papers describing a research protocol without data collection were not included.

**Results:** We analyzed 38 studies. Thirty-six reported a positive effect of the treatment. Seven studies used classical cinematherapy, adopting a qualitative approach to measure the therapy outcome. Thirty-one studies used different video treatments, 8 of which were defined as randomized controlled trials with specific objective therapy outcomes. Studies were mainly focused on behavioral and psychological difficulties in Autism Spectrum Disorders and Schizophrenia.

**Conclusion:** Studies using video treatments more often rely upon structured experimental designs; on the contrary, those who used classical cinematherapy produced descriptive results. A more standardized methodological approach in terms of experimental design, procedure, and objective outcome measure is needed to provide evidence on the effectiveness of these techniques, promoting its application in the clinical field.

## Introduction

Cinematherapy and video treatments are artistic therapeutic techniques in which patients are exposed to complex audiovisual material displaying their psychological or physical difficulties. In the case of cinematherapy, commercial films are used, while in the video treatments, *ad hoc* created videos or brief documentaries are administered to the subjects. Focusing on the patients’ difficulties, the film or video presentation aims to comprehensively understand them through a “third-person” perspective (Berg-Cross et al., 1990). The characters on the screen face the same problems as the patients and offer new ways to cope with them. Such an approach encourages patients to consider their difficulties from a different perspective (Sharp et al., 2002). Typically, the therapist chooses commercial films for an individual to view alone or with specified others. The patient and the therapist look at “reality” with the same magnifying glass by watching a film. This approach also helps strengthen the therapeutic alliance: the therapists’ objective is to allow patients to recognize themselves in one of the characters; thus, they will better understand their emotions and, at the same time, realize their therapists have comprehended their difficulties. Therefore, therapists can better talk about patients’ situations because they have created a “common vocabulary” (Berg-Cross et al., 1990).

Cinematherapy and video treatments originate from bibliotherapy, in which book plots are used with therapeutic aims. These techniques have become increasingly popular with the advent of VHS players, more and more replacing reading books with watching films for therapeutic purposes. Indeed, the use of films or videos is thought to be more “incisive and immediate” (Berg-Cross et al., 1990). One of the first scientific studies on clinical populations dates back to 1974. The authors used videos to treat specific phobias (Morris et al., 1974). Berg-Cross et al. (1990) described the cinematherapy technique providing some guidelines for its clinical application. Later, Heston and Kottman (1997) reported two descriptive cases of patients with depressive disorder treated with cinematherapy. Although using qualitative approaches, these early studies offered promising results, paving the way for other authors to approach the topic more objectively.

Over the years, several authors have also proposed different models of functioning with distinct phases for these techniques. For instance, Dermer and Hutchings (2000) have identified three stages in cinematherapy: (i) *Assessment*, in which the therapist or the counselor identifies the patient’s problems and objectives and chooses films that, in their opinion, fulfill the therapeutic purpose but are also enjoyable to the patient; (ii) *Implementation*, which means that the therapist assigns a film to the patient to let them understand why the therapist has explicitly chosen that film; and then (iii) *Debriefing*, the session after viewing the film for understanding the patient’s reactions. At this stage, the therapist and the patient link the film and the patient’s history (making the cinematherapy effective). Brainstorming is also encouraged to make the patient reflect on what can be therapeutic in the film.

Other authors postulated four main stages for cinematherapy: (i) *Identification*, in which the patient identifies with the character because of the character’s behavior and goals, becoming aware of the character’s feelings and emotions; (ii) *Catharsis*, the patient tries to learn through the character’s experiences; (iii) *Insight*, in which the patient internalizes the character’s experience and creates a connection between the character’s and their experience to become aware of their situation. A further stage was also identified (Jeon, 1992) that is *universalization*, in which the patient does not feel alone because they are watching the character’s story and thus understand how similar they are to the character. This multistage process helps patients act positively toward difficulties in improving problem-solving strategies (Stamps, 2003). In other words, cinematherapy attempts to promote self-exploration and change by using film metaphorically (Berg-Cross et al., 1990).

Using cinema in clinical practice can allow patients to externalize their problems in a less formal context and discuss their problems in a more detached way to overcome their resistance to the therapy (Dermer and Hutchings, 2000). In such a process, emotions and emotional interaction play a pivotal role. One of the main features of the emotional interaction between the seen and the felt is the metaphor (Heston and Kottman, 1997). The film not only has to deal with the patients’ problems clearly to let them create a link with their lives, but it also has to deal with them obscurely so as not to cause resistance that can interfere with the therapy. Consequently, the film must not describe the situation literally; instead, it must do so metaphorically. It generates a triadic relationship between the patient, the therapist, and the film. At this point, a link between the patient’s situation and the situation represented in the film must be created to let the plot enter the patient’s life. Such a metaphor may work on three distinct levels: (i) the literal plot of the film; (ii) the general metaphor beyond the literal plot; and (iii) the patient’s metaphor, in which the film acquires a special meaning based on the patient’s experiences (Heston and Kottman, 1997). Heston and Kottman (1997) helped a woman who had a troubled relationship with her mother by using the film “Lost in Yonkers.” In this specific case, the first level is represented by the literal plot of the film (the story of the two boys who spend the holidays with their grandmother), the second level is represented by the general metaphor (the relationship of a mother with adult children). The third level is the one that represents the patient’s metaphor, the meanings and personal connections that she can find with her own story. When talking about her feelings about the movie, the patient described her experience as follows: *“I was too much like watching my own family [*…*].”*

If, on the one hand, in cinematherapy, commercial films are used as treatment, on the other hand, some researchers create experimental designs using *ad hoc* created videos or documentaries in which some actors show skills of interest to the patient’s case. This technique, called “video modeling,” originated from Bandura’s work (Bandura, 1986). Children naturally imitate social and cultural models to learn skills of interest independent of reinforcement. They can also practice the behavior they have learned in different situations because they have created general schemas to follow. In this process, attention plays an important role: if the subject does not pay sufficient attention to the model, or if the model is not attractive enough, the learning process could be unsuccessful. Moreover, the most salient parts of the process should significantly impact the subject (Taylor and Fiske, 1978; McArthur, 1981). This discovery has led to the creation of videos that show important skills for patients. This technique is typically used with individuals affected by Autism Spectrum Disorder (ASD) and related social difficulties (Walsh et al., 2018), but it can also be used to treat different kinds of pathologies, such as phobias (Morris et al., 1974). The types of video modeling will be explained later in this article.

Cinematherapy and video modeling have been applied in several pathologies, such as Anorexia (Gramaglia et al., 2011), anxiety (Lee et al., 1983), and ASD (Golan et al., 2010; Isong et al., 2014). Many approaches have been used, ranging from traditional cinematherapy (Heston and Kottman, 1997) to *ad hoc*–created video stimuli and documentaries (Brown et al., 2016). Notably, most therapists/researchers do not apply standard criteria to use video stimuli in their studies. They instead adopt an *ad hoc* paradigm or even *ad hoc* video stimuli, cartoons, or documentaries as needed (Golan et al., 2010; Isong et al., 2014), thus preventing any generalizability of the results. Furthermore, not all studies have confirmed their effectiveness. With such a puzzling background, the need for a review of cinema and video therapy is crucial.

### Scoping Review Objectives

The objective of this scoping review is to map the body of literature, describing how researchers and therapists use films or videos to treat psychological/physical difficulties in clinical/subclinical populations and to provide a classification of methodological approaches and outcome measures for future standardization of these techniques.

### Scoping Review Questions

• What is the difference between cinematherapy and video treatment?
• How many studies apply cinematherapy or video treatment to clinical/subclinical populations?
• What kinds of psychological/physical difficulties have been treated?
• What methodological approach has been used (e.g., study design)?
• What outcomes have been measured?
• Was the application of the technique effective?
• What are the major methodological limitations of the available studies?

## Methods

The review was conducted following the PRISMA guidelines for Scoping Review (Tricco et al., 2018). The review included the following four key phases: (i) formulation of the research questions, (ii) research of the effective keywords accordingly to our scope, (iii) selection of the relevant studies in the literature, (iv) relevant information extraction.

### Information Sources and Search Strategy

Studies were selected by querying the following keywords in PubMed, PsycINFO and Google Scholar: “Cinematherapy” or “Cinema therapy” and “Mood” and “Emotion” on 18/10/2019, “Cinematherapy” or “Cinema therapy” and “Mood disorders” on 18/10/2019, “Cinematherapy” or “Cinema therapy” and “Psychiatric disorders” on 18/10/2019, “Cinema therapy” or “Cinema therapy” and “Emotions” and “Mood” and “Psychiatric disorders” on 30/10/2019, “Video peer modeling” and “Cinematherapy” and “Psychiatric disorders treatment” on 30/03/2020, “Video peer modeling” and “Psychiatric disorders treatment,” on 30/03/2020, and “Video treatment” and “Video stimuli treatment” and “Psychiatric disease” on 30/03/2020.

### Eligibility Criteria

Studies included in this scoping review met the following inclusion criteria: (i) research article type, (ii) use of complex visual stimuli including video, films, or documentaries; (iii) application of the techniques on psychological or physical difficulties; (iv) English language.

### Information Extraction

To investigate the suitability of the article, we firstly evaluated the title, then the abstract, proceeding to the entire text. ES and GS agreed on the eligibility criteria of the selected studies. Information of interests such as the type of treatment, the treated pathology, study design, characteristics of the participants’ sample and outcome have been extracted and reported in Table 1. No online protocol is available.

**TABLE 1:** Summary of the analyzed articles.

First author and year of publication	Type of treatment	Treated pathology	Study design	Participants	Outcome
Morris et al. (1974)	Video peer modeling	Snake phobia	Quantitative	*n* = 145 high school students divided into an experimental group (*n* = 66) and control group (*n* = 79).	Positive: The experimental group, compared to the control group, reported lower level of anxiety and avoidance of snakes, as well as a decrease in erroneous beliefs on the subject.
Muzekari (1976)	Video peer modeling	Schizophrenia	Quantitative	*n* = 45 chronic schizophrenic patients, divided into three groups: Experimental group A (Good model; *n* = 15), Experimental group B (Poor model; *n* = 15), Control group (No model; *n* = 15).	Positive: The experimental group that saw positive peer models improved over the controls that saw poor models or no models.
Lee et al. (1983)	Video treatment	Agoraphobia	Quantitative (Randomized control trial)	*n* = 32 Agoraphobic patients were randomly divided into three experimental groups and a control group (*n* = 8 for each group).	Positive: The “faded” group showed significant decreased phobic behavior than the controls and the supraliminal group. The improvement was maintained after 12 weeks.
Charlop and Milstein (1989)	Video modeling	Autism spectrum disorder (ASD)	Quantitative (Multiple baseline design)	*n* = 3 children with ASD, no control group.	Positive: Children learned new conversational skills from videos and generalized them to other situations as well. They kept the skills even 15 months apart.
Turley and Derdeyn (1990)	Classical cinematherapy	Violent behavior	Quantitative (Single case description)	*n* = 1 boy	Positive: Discussion with the therapist about the thoughts, concerns, and motives of the characters in the horror films allowed the boy access to his preconscious conflicts.
Gelkopf et al. (1993)	Video treatment	Schizophrenia	Quantitative	*n* = 34 patients with Schizophrenia were divided into an experimental group (Humorous movie, *n* = 17) and a control group (Other movies, *n* = 17)	Unclear: No significant improvement was reported by the two groups of patients. Nevertheless, the psychiatric staff reported a significant reduction in negative emotional states in the patient and an improvement in the degree of perceived staff support. Results may be due to an incidental positive effect on staff emotional state.
Heston and Kottman (1997)	Classical cinematherapy	Depression	Qualitative	*n* = 2 patients with depression. No control group.	Positive: Patients gained new insights about their condition and improved in therapy.
Thiemann and Goldstein (2001)	Video feedback	Autism spectrum disorder (ASD)	Quantitative (Multiple baseline design)	*n* = 15 children divided in ASD group (*n* = 5) and Neurotypical group (*n* = 10).	Positive: Children improved their conversation skills and generalized them to other contexts.
Bierman et al. (2003)	Classical cinematherapy	Different disorders concerning mood and behavior	Qualitative	*n* = 15 adolescent girls with different diagnoses. No control group.	Positive: The girls were able to relate to the films and successfully discuss them, improving their therapeutical process.
Gelkopf et al. (2006)	Video treatment	Schizophrenia	Quantitative (Comparative study)	*n* = 29 patients with Schizophrenia divided into one experimental group (*n* = 15) and a control group (*n* = 14).	Positive: Reduction of negative symptomatology, decrease in negative emotions and improvement in social skills.
Kimata (2007)	Video treatment	Atopic dermatitis and Night awakenings	Quantitative	*n* = 80 divided into *n* = 40 children with Atopic dermatitis and Night awakenings and *n* = 40 healthy children.	Positive: Humorous films decreased nocturnal awakenings and ghrelin levels in the saliva of children with dermatitis, while no significant effect is recorded for the healthy ones.
Reeve et al. (2007)	Video modeling	Autism spectrum disorder (ASD)	Quantitative (Multiple baselines across participant design)	*n* = 4 children with ASD. No control group.	Positive: Children learnt to address correct responses for helping behaviors and to generalize responses to other situations. They maintained the new skills after a 60 days follow-up.
Scattone (2008)	Video modeling	Asperger syndrome	Quantitative (Multiple baseline design, case study)	*n* = 1 adolescent with Asperger. No control group.	Positive: Improvement in 2 of the 3 target skills, as well as a generalization of the same skills
Kimata (2008)	Video treatment	Atopic dermatitis and Erectile disfunction	Quantitative (Randomized controlled trial)	*n* = 36 men with atopic dermatitis suffering from erectile dysfunction divided into one group (*n* = 18 men watching humorous films first), another group (*n* = 18 men watching control films first)	Positive: When participants were exposed to humorous films, erectile dysfunctions diminished, testosterone increased, and estradiol levels decreased, while control films failed to obtain the same outcome.
Coughlin et al. (2009)	Video modeling	Behavioral problems	Quantitative (Controlled clinical trial)	*n* = 74 parents of children with behavioral problems divided into Enhanced video-based treatment group (*n* = 42); Treatment as usual (TAU) comparison group (*n* = 32).	Positive: Both immediately after the end of treatment and 5 months after treatment, the Parents Plus Children’s Programme (PPCP) group decreased conduct problems and problems with peers (concerning children), then decreased parental distress and increased parental self-esteem. Parents’ ability to define problems and goals has increased. Treatment is most effective for children who only have behavioral problems. The positive changes were maintained to a 5-months follow-up.
Golan et al. (2010)	Video treatment	Autism spectrum disorder (ASD)	Quantitative (Comparative study)	*n* = 56 children divided into an experimental group (*n* = 20 children with ASD administered with the video-based intervention), ASD control group (*n* = 18 children with ASD without video intervention), Normally developing children group (*n* = 18 neurotypical children)	Positive: Experimental subjects significantly improve their performance, reaching a performance comparable to both control groups.
Marsick (2010)	Classical cinematherapy	Parental divorce	Qualitative (Collective case study)	*n* = 3 children whose parents were divorcing. No control group.	Positive: The children were able to reflect better on the situation and increased their awareness of the situation.
Lim (2010)	Video treatment	Autism spectrum disorders (ASD)	Quantitative (Randomized controlled trial)	*n* = 50 children with ASD divided into a music condition group (*n* = 18), Speech condition group (*n* = 18), and Control group (*n* = 14)	Positive: Children in both the music and speech groups improved significantly in their speech, those with low functioning showed greater improvement after music training.
Marx et al. (2010)	Video treatment	Dementia	Quantitative	*n* = 56 patients with dementia. No control group	No effect: Patients can be positively engaged with dog-related stimuli, particularly with real dogs. No significant differences were found in engagement duration among our dog-related stimuli.
Corbett et al. (2011)	Video peer modeling	Autism spectrum disorder (ASD)	Quantitative (Pre-test-post-test design)	*n* = 8 children with ASD. No control group.	Positive: ASD participants showed an improvement in the Theory of Mind and face recognition.
Gramaglia et al. (2011)	Classical cinematherapy	Anorexia Nervosa	Qualitative (Single-case study)	*n* = 1 woman with binge-purging anorexia nervosa	Positive: The treatment increased the patient’s awareness of her pathological condition and allowing a better tolerance of psychotherapy treatment, with positive repercussions on the patient’s daily life
Perlick et al. (2011)	Video peer modeling	Schizophrenia	Quantitative (Randomized controlled trial)	*n* = 122 caregivers of patients with schizophrenia divided into a Peer-led intervention group (*n* = 59), and a Clinical-led intervention group (*n* = 63).	Positive: Caregivers receiving peer-led video-based intervention experienced marked improvement in self-stigma and secrecy.
Rayner (2011)	Video prompting	Autism spectrum disorder (ASD)	Quantitative (Case reports)	*n* = 3 children with ASD who attend to both video prompting and backword chaining technique.	Unclear: Although the video prompting interventions increased the number of steps in the shoelace tying task completed by each participant, the backward chaining procedure was more effective, enabling one participant to reach mastery and a second participant to approach mastery.
Wilkes et al. (2011)	Video self-modeling	Attention deficit and Hyperactivity disorder	Quantitative	*n* = 28 children divided into an attention deficit and Hyperactivity disorder (ADHD) group (*n* = 14), and a Typical developing children group (*n* = 14).	Positive: Both children with ADHD and peers have improved their social play skills.
Ballard (2012)	Classical cinematherapy	Relationship problems	Qualitative (Case study)	A couple with relationship problems. No control group	Positive: The film helped participants become aware of the nature of their problems and speak positively about them.
Olatunji et al. (2012)	Video treatment	Blood-injection-injury phobia	Quantitative (Randomized controlled trial)	*n* = 44 subjects with blood-injection-injury phobia divided into an experimental condition group (*n* = 22 exposed to “disgusting” condition), and a Neutral condition group (*n* = 22 exposed to neutral videos).	Positive: The subjects who viewed the “disgust-condition” videos felt more disgust than those who saw the neutral videos. All the participants exposed to the videos with sampling images improved at the end of the treatment.
Savorani et al. (2013)	Video treatment	Dementia	Quantitative	*n* = 20 patients with dementia divided into an experimental group (*n* = 10 patients undergoing video-treatment), and a Control group (*n* = 10 patients with usual treatment).	Positive: There was a decrease in NPI scores and the distress levels of attendants and relatives.
Isong et al. (2014)	Video peer modeling and video treatment	Autism spectrum disorder (ASD)	Quantitative (Randomized controlled trial)	*n* = 69 children with ASD and dental fear divided into a video peer modeling only group (*n* = 17), Video goggles only group (*n* = 15), Video goggles plus video peer modeling only group (*n* = 18), and a Control group (*N* = 19).	Positive: The video goggles group and the peer modeling group plus video goggles have improved follow-up visits (4–6 months).
Jones et al. (2014)	Video modeling	Autism spectrum disorder (ASD)	Quantitative (Concurrent multiple baseline design)	*n* = 4 children with ASD.	Positive: Results suggested that certain irrelevant stimuli (adult vs. peer recipient) were more likely to exert stimulus control over responding than others (setting, materials) and that video viewing was an efficient way to promote generalization to peers.
Decker and Buggey (2014)	Video self-modeling vs. video peer modeling	Learning disabilities	Quantitative (Multiple baselines across participant design)	*n* = 9 children with learning disabilities divided into a video peer modeling group (*n* = 3), a Self-modeling group (*n* = 3), and a Control group (*n* = 3).	Positive: Both video self-modeling and video peer modeling make the children improve reading fluency. Positive effects were maintained at follow-up (6 weeks for the first group, 4 for the second and 2 for the third).
Macpherson et al. (2015)	Video modeling	Autism spectrum disorder (ASD)	Quantitative (Multiple baselines across participant design)	*n* = 5 Children with ASD. No control group.	Positive: Subjects increased the use of compliments and expanded the use of responses. These behaviors have also been generalized to other situations.
von Maffei et al. (2015)	Video modeling	Schizophrenia	Quantitative (Quasi-experimental pre-post design)	*n* = 113 patients with schizophrenia. No control group.	Positive: Subjects increase their knowledge of disease, insight, and improve for quality of life. The improvements could still be observed after a year.
Copple et al. (2015)	Video modeling	Autism spectrum disorder (ASD)	Quantitative (Single-subject multiple baseline design)	*n* = 3 Children with ASD or Autism symptoms. No control group.	Positive: All three participants demonstrated the ability to request preferred objects following the intervention and generalize the newly acquired behavior across stimuli and people.
Brown et al. (2016)	Video peer modeling	Smoking	Quantitative (Randomized controlled trial)	*n* = 3,019 smokers divided into no-intervention control group (*n* = 1,016), Informative intervention group (*n* = 1,004), and an experimental treatment group (*n* = 999).	No effect: There was no difference between the subjects undergoing the experimental treatment, the informative treatment, and those who did not receive any treatment.
Eğeci and Gençöz (2017)	Classical cinematherapy	Relationship problems	Qualitative (Descriptive study)	*n* = 6 women with relationship problems. No control group.	Positive: the viewing step itself did not promote change; instead, the discussion phase induced new insights and facilitated the generalization of these gains into individuals’ problem areas.
Walsh et al. (2018)	Video modeling	Autism spectrum disorder (ASD)	Quantitative (Multiple-probe design)	*n* = 7 young adults with ASD. No control group	Positive: Results showed significant increases in target social skills and a significant decrease in problem behaviors following the intervention. Evidence of maintenance and generalization was also demonstrated up to the 3-month follow-up.
Yan et al. (2018)	Video treatment	Autism spectrum disorder (ASD)	Quantitative	*n* = 21 children divided into an ASD group (*n* = 14 children with ASD), and a Control group (*n* = 7 typically developing children).	Positive: The intervention improved ASD children’s emotion recognition compared to their pre-intervention scores.
Dueñas et al. (2019)	Video Joint modeling	Autism spectrum disorder (ASD)	Quantitative (Multiple Probe Cross participant design)	*n* = 6 children divided into a patient group (*n* = 3 children with ASD), and a control group (*n* = 3 typically developing children).	Positive: The participants improved unscripted verbalizations during pretend play with typically developing peers in an inclusive early childhood setting. Moreover, participants learned to use verbalizations even not included among those taught during the treatment, and these remained even in the absence of the video models.

## Results

**FIGURE 1:**
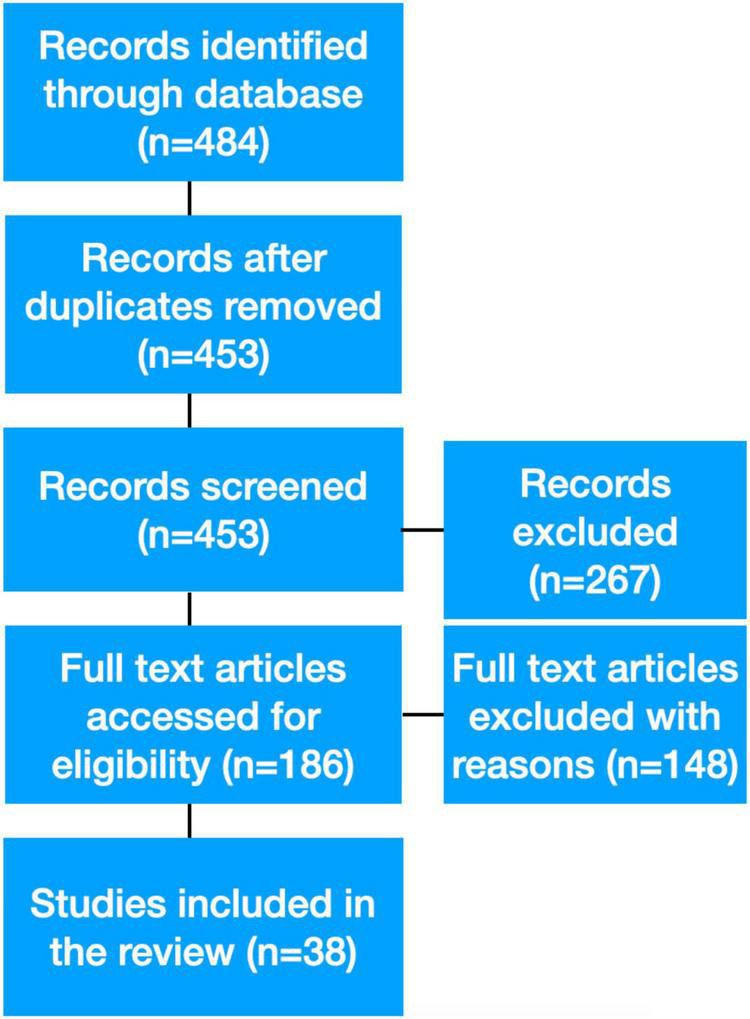
The flow chart shows the selection process of the articles.

Through databases, 484 articles were identified, and after duplicates were removed, 453 articles were analyzed. After analyzing the content of 186 articles, 148 were excluded because they did not meet the inclusion criteria, such as studies in which cinema or videos were not used for therapeutic aims. Thirty-eight studies published from 1974 to 2018 comprised the final sample included in the review (Figure 1). Among studies included in this review, seven used classical cinematherapy (Turley and Derdeyn, 1990; Heston and Kottman, 1997; Bierman et al., 2003; Marsick, 2010; Gramaglia et al., 2011; Ballard, 2012; Eğeci and Gençöz, 2017), 9 used video modeling approach (Charlop and Milstein, 1989; Reeve et al., 2007; Scattone, 2008; Coughlin et al., 2009; Jones et al., 2014; Copple et al., 2015; Macpherson et al., 2015; von Maffei et al., 2015; Walsh et al., 2018), 5 applied video peer modeling (Morris et al., 1974; Muzekari, 1976; Corbett et al., 2011; Perlick et al., 2011; Brown et al., 2016), 1 used video self-modeling, (Wilkes et al., 2011), one combined video peer modeling and video self-modeling (Decker and Buggey, 2014). Eleven out of 38 studies used a generic video treatment (Lee et al., 1983; Gelkopf et al., 1993, 2006; Kimata, 2007, 2008; Golan et al., 2010; Lim, 2010; Marx et al., 2010; Olatunji et al., 2012; Savorani et al., 2013; Yan et al., 2018), 1 with video feedback (Thiemann and Goldstein, 2001), 1 with video prompting (Rayner, 2011), 1 with video peer modeling and a generic video treatment (Isong et al., 2014), and 1 with video joint modeling (Dueñas et al., 2019). Notably, 36 of the 38 studies reported a positive effect of the technique. All the articles applying classical cinematherapy can be classified as qualitative studies. In the case of video treatments, a quantitative approach has been mainly used. For a more detailed characterization of each study (see Table 1).

In the following paragraphs, we define cinematherapy and video techniques arising from the scoping review of the literature. We also summarized their features, such as the type of film stimuli used, the type of treatments applied, and the populations to which these treatments are administered.

### Types of Treatment

In this section, we will summarize the most common ways to use films or videos to treat people (classical cinematherapy and video modeling), and we will show some examples of video treatment.

#### Traditional Cinematherapy

Traditional cinematherapy can be defined as a therapeutic technique in which the therapist selects commercial films and asks the client to see them alone or with specific other people (e.g., family members). In particular, the therapist chooses some films that, in their opinion, relate to the patient’s difficulties and, after a viewing session, discusses with the patient the main themes that emerged from the movie. For example, Ballard (2012) showing *Shrek 2*, a cartoon in which a friend of the two protagonists impedes in their life as a couple, helped a couple become aware that the problems of their relationship were due to their intrusive parents and friends. Another interesting reflection is that viewing specific films can be effective, even when patients accidentally see them (Heston and Kottman, 1997). The therapist must find a film with the characteristics described above that matches the subject’s preferences, goals, interests, and comprehension ability (Heston and Kottman, 1997). With this in mind, it is desirable to use films with content that the patient can endure; for example, showing a patient a film in which their problem is treated overly explicitly and traumatically can be counterproductive (Heston and Kottman, 1997). Moreover, if the patient is in a particular phase of therapy, such as working hard to change, the therapist should avoid cinematherapy (Berg-Cross et al., 1990).

#### Video Modeling

The video modeling technique exposes patients to videos in which characters manifest specific skills that the patients need to learn (e.g., learning how to interact with other people successfully). At the end of the video, the patients can practice what they learned from the character. It is also important to note that video modeling techniques are sometimes combined with other treatments (e.g., psychotherapy) (Corbett et al., 2011; Perlick et al., 2011).

In a clinical setting, video modeling has mainly been used to improve social skills in individuals affected by ASD, ranging from the appropriate behavior in a workplace environment (Walsh et al., 2018) to the ways of initiating and carrying on a social conversation during daily activities (Charlop and Milstein, 1989; Thiemann and Goldstein, 2001; Scattone, 2008; Dueñas et al., 2019), and from positive behavior when playing with peers (Macpherson et al., 2015) to helping behavior (Reeve et al., 2007).

Another application of video modeling exposes patients to videos of people recounting their experiences of a particular event (Perlick et al., 2011; Brown et al., 2016). For example, Brown et al. (2016) study screened a documentary that portrayed five smokers who shared their experiences in the first month of their successful attempts to stop smoking, day by day, like in a diary.

In addition to the above-presented general structure of video modeling, this technique can be employed in different ways using various labels. An example is *video prompting*, which provides a step-by-step learning experience. In video modeling, all the skill passages (e.g., tying shoelaces) are usually shown together and followed by the option to attempt the skill. Unlike typical video modeling, video prompting shows each section of the video separately so that the subject can try each step of the skill (Rayner, 2011). *Joint video modeling* pairs a joint exposition with a video, and the subject interacts with a peer without the target pathology and watches the same video while receiving the same treatment (Dueñas et al., 2019).

If the characters in the videos are the patient’s peers, then the treatment is called *video peer–modeling*. This model shares particular patient features, such as age (Isong et al., 2014) or pathology (von Maffei et al., 2015; Brown et al., 2016). Watching the main character in the video offers a way of coping with difficulty because it encourages patients to adopt new strategies for their problems. For example, Morris et al. (1974) used four 12–min color films. The videos were categorized as experimental and control stimuli. Their content was as follows: (i) a student interacting with a female herpetologist and learning to manipulate snakes ranging from little ones in a cage to bigger ones in her hands (experimental) while a male narrator explains the scene and provides information about snakes; (ii) the same film except for an additional scene in which the protagonist lets the big snake winds around her neck (experimental); (iii) a snake moving in a cage (without any explanation), and (iv) some beautiful scenarios with no references to snakes.

Another variant of video modeling is *self–video modeling*, in which the target subject acts as a self-model instead of using an external model (an actor or a peer). The therapist video records every treatment session and follows up by showing these videos to the participant, who then reflects on the target behavior (Wilkes et al., 2011).

Video modeling is also used for developing psychological treatments. The Parents Plus Children Programme (Sharry and Fitzpatrick, 1998), for example, was created to help parents of children with behavioral problems. It consisted of videos of both excellent and inadequate management of problematic situations, and the characters were both real parents and actors. After viewing the film, the people who took part in the treatment discussed its contents. In 2009, Coughlin et al. (2009) retested the treatment on parents of children from 6 to 11 years old with and without other pathologies besides behavioral problems (for example, from ASD to learning or language problems) and found a decrease in behavioral problems, stress levels, with augmented trust, and parent-defined goal achievement. At a 5 months follow-up, the participants maintained these skills and showed an improvement in prosocial behavior.

#### Additional Use of Videos in Treatment

This paragraph includes some examples of other ways to use video or cinema to treat different pathologies. For instance, Lee et al. (1983) have treated patients with agoraphobia using specific manipulated videos that gradually expose patients to the target of their phobias. Patients were informed that the treatment consisted of watching films for 4 weeks and a follow-up after 3 months. Patients were assigned to one of four conditions in random order: (i) subliminal stimuli, (ii) supraliminal stimuli, (iii) faded stimuli, and (iv) control condition. Every subject in the subliminal vision, faded vision, and control conditions saw the stimuli, a spot of light varying in intensity, under the threshold level. In the faded condition, the filters were positioned so that the stimulus changed from entirely subliminal to entirely supraliminal. Accordingly, the experimental group saw stimuli concerning agoraphobia, and the control group saw films in which a man constructed a clay pot. Similarly, Olatunji et al. (2012) have studied the effect of habituation on Blood Injection Injury phobia through exposure to threatening videos.

In another setting, Kimata (2007, 2008) screened humorous films for patients affected by atopic dermatitis and other manifestations of this stress-related illness. In an initial study Kimata (2007) found a diminished level of ghrelin in the saliva of patients suffering from atopic dermatitis and frequent night awakening in children suffering from it. In a second study, the author (Kimata, 2008) demonstrated that this treatment might diminish estradiol levels and increase testosterone levels in men affected by atopic dermatitis and erectile dysfunction. According to the author, exposure to comic films can reduce stress effects and improve the symptoms of the pathology connected with it (Kimata, 2007, 2008).

Further applications of humorous films come from Glekopf’s study (Gelkopf et al., 2006). The authors applied this technique to people affected by schizophrenia. The treatment consisted of daily exposure to comedies 5 days per week for 3 months. Results showed a reduction in patients’ anxiety and depression levels. Nevertheless, the mere vision of the film is not enough to make the patient improve, so the therapist must use the “relaxation” created by the film for traditional psychological therapy, as we can see from the same author’s study of 1993 (Gelkopf et al., 1993).

Cartoons are also effective for the video treatment. Golan et al. (2010) used a specific cartoon in which “repetitive vehicles,” such as trains, were presented. The trains were depicted with a human face expressing 15 target emotions to teach children to recognize them. This study was conducted to train children with ASD to improve their ability to recognize emotions by looking at facial expressions. The study was also replicated by Yan et al. (2018).

Lastly, the music video, the speech video, and no treatment were compared to investigate the most effective method for improving the conversational skills of children with ASD (Lim, 2010). Results have shown that songs (containing specific keywords with a technique called Developmental Speech and Language Training Through Music created *ad hoc* and speech videos with the same content as the songs improved language skills in children with high functioning ASD. Children with low-functioning ASD, however, only benefited from music videos.

### Outcome Measures

Different outcome measures have been employed to explore changes due to video exposure. Often, cinematherapy does not involve quantitative analysis (pre-post); researchers simply discuss the movie content with the patients during and after the session, sometimes in combination with qualitative questionnaires (Eğeci and Gençöz, 2017).

#### Psychological Tests

Outcomes are frequently measured using psychological tests. For example, Lee et al. (1983) study used psychological tests to assess phobias, avoidance behaviors, depression, panic attacks, and anxiety. Simultaneously, an external operator (ignoring that the subject was undergoing an experimental assessment) investigated the same themes by interviewing participants. Isong et al. (2014), study though, the Venham Anxiety and Behavioral Scale, a measure of anxiety and behavioral problems, was used at the end of the visits.

#### Biological Markers

Studies have also assessed physiological parameters according to their aims when participants suffer from an organic illness or when the phenomenon under consideration involves physical parameters. For instance, in a study assessing the effects of viewing humorous films on erectile dysfunction in patients with atopic dermatitis, Kimata (2008) collected serum testosterone and estradiol levels. In another study, Kimata (2007) collected the saliva of children with atopic dermatitis and measured ghrelin levels and night-awakening events.

The authors also measure physiological parameters if it is helpful to understand psychological changes, as is the case with anxiety. For example, Lee et al. (1983) conducted a study on agoraphobia using subliminal, supraliminal, and faded stimuli. Participants completed bodily symptoms ratings after each stimulus exposure (Tyrer and Lader, 1973) and mood (Bond and Lader, 1974) ratings. They were asked about muscular tension, heart rate, sweatiness, breathing difficulties, shaking, relaxation, and excitement. However, skin conductance, respiratory rate, and heart rate were measured during the film to obtain objective data.

### Clinical Applications

Cinematherapy and video modeling can treat various psychological difficulties and particular conditions in healthy and pathological populations. For instance, Marsick’s (2010), study children who experienced parental divorce increased their awareness of their feelings and the situation after watching films about separation and divorce. Specifically, the results showed that people preferred films specifically conceived for their ages and historical periods. In another study (Bierman et al., 2003), cinematherapy was used to treat young girls with legal and family problems, demonstrating enhanced compliance and cooperation as well as an understanding of their family problems.

Other examples of pathologies in which video modeling is applied are ASD (Charlop and Milstein, 1989; Lim, 2010; Isong et al., 2014; Copple et al., 2015; Macpherson et al., 2015), addiction (Brown et al., 2016), schizophrenia (Muzekari, 1976; von Maffei et al., 2015), anxiety (Morris et al., 1974), dementia (Marx et al., 2010; Savorani et al., 2013), and anorexia nervosa (Gramaglia et al., 2011). It is also used for caregivers (Perlick et al., 2011) and people with learning disabilities (Decker and Buggey, 2014).

## Discussion

Cinematherapy and video treatments can help people manage life challenges. Movies or videos can help develop new skills, increase awareness, and offer new ways of thinking about specific problems in various patient populations. Cinematherapy and video treatments are flexible: they can be applied in several modalities and are adaptable to diverse situations. Nevertheless, there are some critical concerns. In classical cinematherapy, the choice of viewing material is not standardized; it varies according to the therapist’s beliefs and experience. Even if some therapists have proposed lists of films that can be utilized to treat different emotional difficulties (Berg-Cross et al., 1990), these simply remain as advice.

This scoping review found that 38 articles using classical cinematherapy and others employing video modeling (or its subcategories) or other forms of video treatment have been applied to clinical/subclinical populations. In most cases (14 studies), the video technique was applied to treat behavioral difficulties related to ASD. Interestingly, we also found five studies that examined the effect of cinematherapy and video treatments in patients affected by Schizophrenia.

Authors who used video treatments or video modeling are more inclined to implement experimental designs; on the contrary, those who used classical cinematherapy produced descriptive studies. Indeed, 8 of the 38 studies were designed as randomized controlled trials and all involved video treatment as a technique. This finding could be intrinsic to the differential nature of cinematherapy and video treatments. On the one hand, cinematherapy uses video material that is strongly characterized by an artistic component and thus challenging to standardize; on the other hand, video treatment involves straightforward video material without artistic intent. When cinematherapists use a film, they manage a complex artistic medium to treat patients. Transforming this approach into a standardized paradigm is far from banal. In some cases, standardizing a specific protocol appears to be more of a limitation than a treatment improvement. Due to these methodological limitations, estimating the objective efficacy for cinematherapy is complex, and the results, therefore, remain mainly descriptive.

Another issue involves a possible bias on the outcome effect: 36 out of 38 studies reported that the treatment had a positive effect. Consequently, a positive-results bias should also be taken into account. In one study that showed the technique to be ineffective, the authors ran a randomized controlled trial exploring the possibility of helping people quit smoking through videos (Brown et al., 2016). Brown et al. (2016) administered an online documentary film to motivate quit attempts among smokers in the general population. In this study, participants were randomly exposed to one of the three conditions: no-intervention control (*n* = 1,016), an informational film (*n* = 1,004), or an *ad hoc* documentary (*4 Weeks2Freedom*) (*n* = 999). Results showed that there was no detectable effect of the intervention compared with the no-intervention control or informational control film. Among the possible causes of the negative result, the authors suggested that the film may not have been sufficiently engaging to keep participants viewing for long enough to have an effect. Furthermore, they stated that the intervention was unsuccessful as the identification process according to which viewers identify with the people in the *ad hoc* documentary might not have been effective. We also advise caution when using video modeling, as its efficacy may also depend on the choice of material. For instance, in some studies, videos with adult models were shown to children to improve their skills (Charlop and Milstein, 1989). However, Jones et al. (2014) found that when social skills were taught to children with ASD using adults as models, the children only applied the new competencies when interacting with other adults, not with peers. Finally, it is interesting to note that Isong et al. (2014) suggested that this treatment is effective only when combined with additional instruments (e.g., video peer modeling plus video goggles).

Another critical aspect of this technique is the pathology type for which it is applied. Whereas researchers use classical cinematherapy or video treatment for a large plethora of difficulties (from children with divorced parents to blood phobia), they more often successfully use video modeling to treat people with ASD (14 studies). As a result, we cannot have a global view of the actual efficacy of this technique for other kinds of pathologies. It would be interesting to apply it to different patient populations and other psychological/physical difficulties to analyze who can best benefit from the treatment.

The main criticism of using cinematherapy and video treatment is that they lack a standardized set of stimuli and protocols. Future studies should aim to create standardized protocols, above all in cinematherapy practice, for patient populations. It would be helpful to select a film or some particular scenes of a film and standardize them through an adequate number of healthy subjects who judge the emotional valence of the selected stimuli. Most importantly, the main outcome should be measured objectively. A rigorous methodological approach will allow for the scientific validation of these audiovisual techniques, which are easy to administer and flexible.

We have all been to the cinema and have relived the emotions we were experiencing through the characters in the film. This experience is a widespread testimony to the value of the technique. Nevertheless, its justification cannot remain purely anecdotal; further scientific validation is necessary.

### Limitations

One of the limitations of this study concerns the detection of published papers on the topic. In fact, in some cases, studies on art therapy are published in journals that are not indexed in major search engines such as PubMed. For this reason, this review may not have taken these papers into account. To deal with this issue, we attempted through cross-searching to include all published works in which cinematherapy and video treatments were applied to clinical and subclinical populations. Lastly, because the review was limited to papers published in the English language, it is possible that other potentially relevant papers were omitted.

## Conclusion

To conclude, the mapping of the published literature shows that a rigorous and robust methodology is not characteristic of diverse approaches, making the generalization of the results, if anything, more complicated. Cinematherapy and video treatments are flexible, easy-to-use approaches that can be applied to different patient populations. Although the application of stricter rules is complicated due to the complexity of the adopted stimuli, we believe that such an effort is desirable: implementing standard protocols with more precise efficacy indexes will make these treatments objectively valid rehabilitation tools.

## Significance Statement

The art therapy landscape is vast and complex. In recent years, the application of cinematherapy and video modeling to treat psychological difficulties has increased enormously. Nevertheless, the methodological approach is mixed, and the outcome measures are mainly subjective. To our knowledge, this scoping review represents the first attempt to map the available studies on cinematherapy and video treatment, with a twofold implication. First, it organizes what we know so far about cinematherapy and video treatment applied to clinical populations. On the other hand, it represents a starting point from which future research can improve protocols, increasing the efficacy of the use of these techniques at the intersection of art and psychology.

## Data Availability Statement

The original contributions presented in the study are included in the article/supplementary material, further inquiries can be directed to the corresponding author/s.

## Author Contributions

ES: investigation, data curation, and writing—original draft. GS: conceptualization, writing—original draft, and writing—review and editing. FS and FV: writing—review and editing. GB: conceptualization, writing—review and editing, and supervision. All authors contributed to the article and approved the submitted version.

## Conflict of Interest

The authors declare that the research was conducted in the absence of any commercial or financial relationships that could be construed as a potential conflict of interest.

## Publisher’s Note

All claims expressed in this article are solely those of the authors and do not necessarily represent those of their affiliated organizations, or those of the publisher, the editors and the reviewers. Any product that may be evaluated in this article, or claim that may be made by its manufacturer, is not guaranteed or endorsed by the publisher.
